# Supplementary material for: Phosphorylation of Thymidylate Synthase and Dihydrofolate Reductase in Cancer Cells and the Effect of CK2α Silencing
Source: Int J Mol Sci. 2023 Feb 3;24(3):3023. doi: 10.3390/ijms24033023 (PMC9917831; doi:10.3390/ijms24033023)
Supplement: Supplementary file 1 [file ijms-24-03023-s001.zip › ijms-2173739-supplementary.pdf]

Supplementary material

Phosphorylation of Thymidylate Synthase and Dihydrofolate Reductase in Cancer Cells and the Effect of CK2α Silencing

Patrycja Wińska [1,*], Anna Sobiepanek [1], Katarzyna Pawlak [2], Monika Staniszewska [3,*] and Joanna Cieśla [1]

[1] Chair of Drug and Cosmetics Biotechnology, Faculty of Chemistry, Warsaw University of Technology, 00-664 Warsaw, Poland
[2] Chair of Analytical Chemistry, Faculty of Chemistry, Warsaw University of Technology, 00-664 Warsaw, Poland
[3] Centre for Advanced Materials and Technologies, Warsaw University of Technology, Poleczki 19, 02-822 Warsaw, Poland
* Correspondence: patrycja.winska@pw.edu.pl (P.W.); monika.staniszewska@pw.edu.pl (M.S.); Tel.: +48-222-345-573 (P.W.); +48-606-438-241 (M.S.)

1. TS and DHFR preparation results

The purified recombinant TS and DHFR were co-polymerized with the gel and subsequently were used in an in-gel kinase assay.

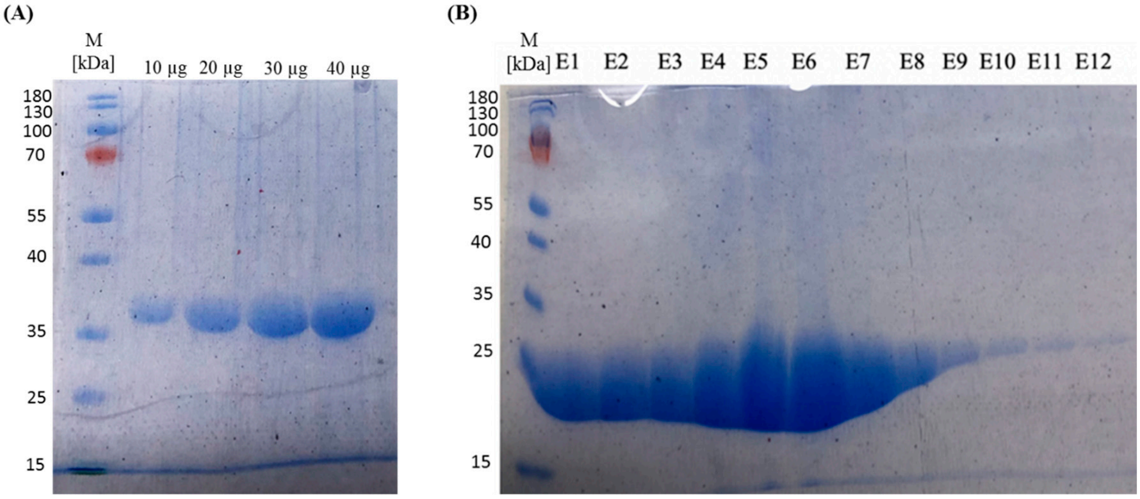

Figure S1. SDS-PAGE of the TS (A) and DHFR (B) preparations obtained after NiNTA-Agarose affinity chromatography, which subsequently were used in the in-gel kinase assay. The gels were stained with CBB after SDS-PAGE of the eluted fractions of the purified recombinant enzymes. (A) 10-40 µg TS was obtained after combining the eluted fractions. (B) E1-E12 fractions of DHFR.

2 In-gel kinase assay results

We have observed that some parts of co-polymerized enzymes migrate during SDS-PAGE according to their molecular weight, i.e., 36 kDa and 21 kDa for TS and DHFR, respectively (Figure S2).

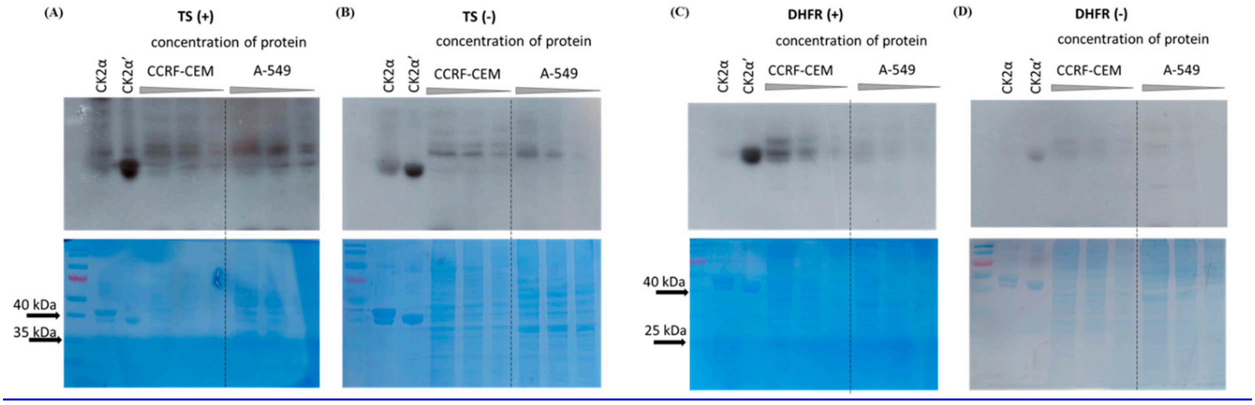

Figure S2. In-gel kinase assay results for TS (A) and DHFR (C). CCRF-CEM and A-549 lysates were prepared in NP-40 buffer. Human recombinant catalytic subunits of CK2, i.e. CK2*α* (29 μg) and CK2*α*′ (8 μg) were used as controls. Three different amount of protein were loaded of each lysate in the range from 89-22 μg/well. Upper part of the picture are autoradiograms (after 18 h and 6 h of X-ray films exposure for TS and DHFR, respectively) and the lower part of the pictures represent dried gels stained with CBB (B,D). Control gels without substrates represent autophosphorylation of protein kinases in lysates.

3 Identification of proteins in the excised gel band by LC-MS/MS

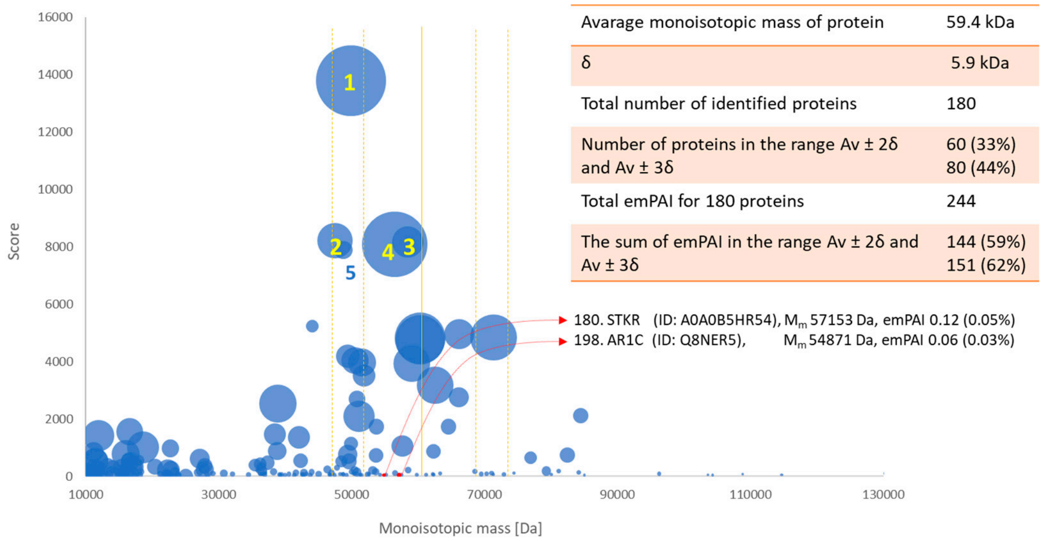

Figure S3. The molecular weight distribution of the proteins in the excised gel band against the Score describes the probability of matching experimental data with proteins in the database. The bubble size corresponds to the emPAI value – the parameter dependent on the protein concentration. Most abundant proteins are indicated with numbers: 1. keratin, 2. α-enolase, 3. α-amylase, 4. keratin, type II cytoskeletal 4, 5. keratin, type I cytoskeletal.

Table S1. Characteristics of peptides detected by LC-MS/MS matching sequence of CK2α and CK2α′ proteins

Sequence	Observed m/z	Charge (z)	Position of sequence start - end	Experimental Mm [Da]	Theoritical Mm [Da]	ΔM [ppm]	Coverage (y, a-b daughter ions) [%]	Score (> 37)	p < 0.05	Unique to protein family
Peptides specific to casein kinase II subunit alpha										
FNDILGR	417.7263	2	269 - 275	833.438	833.4395	-1.83	67	41	0.00026	YES
GGPNIITLADIVK	655.8885	2	90 - 102	1309.7625	1309.7605	1.5	100	90	2.3e-09	YES
VYTDVNTHRPR	340.1791	4	11 - 21	1356.6874	1356.6898	-1.79	35	45	0.00028	YES
VYTDVNTHRPR	453.2368	3	11 - 21	1356.6886	1356.6898	-0.91	55	53	4.6e-05	YES
VYTDVNTHRPR	679.3528	2	11 - 21	1356.691	1356.6898	0.88	65	42	0.00049	YES
QLYQTLTDYDIR	764.8856	2	123 - 134	1527.7566	1527.7569	-0.17	59	68	1.4e-06	YES
TPALVFEHVNNTDFK	577.962	3	108 - 122	1730.8642	1730.8628	0.82	71	79	1.1e-07	YES
TPALVFEHVNNTDFK	866.4395	2	108 - 122	1730.8644	1730.8628	0.91	79	127	1.9e-12	YES
LIDWGLAEFYHPGQEYNVR	769.7118	3	173 - 191	2306.1136	2306.112	0.69	42	105	4.0e-10	YES
LIDWGLAEFYHPGQEYNVR	769.7119	3	173 - 191	2306.114	2306.112	0.86	42	101	1.0e-09	YES
FVHSENQHLVSPEALDFLDK	775.7216	3	284 - 303	2324.1428	2324.1437	-0.38	68	63	6.8e-06	YES
VLGTEDLYDYIDKYNIELDPR	848.7554	3	248 - 268	2543.2444	2543.2431	0.5	63	111	1.0e-10	YES
Peptides specific to casein kinase II subunit alpha'										
VYAEVNSLR	525.7829	2	12 – 20	1049.5513	1049.5505	0.72	63	65	2.1e-06	YES
HLVSPEALDLLDK	725.4022	2	292 - 304	1448.7898	1448.7875	1.62	83	92	3.8e-09	YES
HLVSPEALDLLDK	725.4032	2	292 - 304	1448.7918	1448.7875	2.98	67	78	1.1e-07	YES
QLYQILTDFDIR	762.9068	2	124 - 135	1523.799	1523.7984	0.43	68	84	3.2e-08	YES

ΔM – monoisotopic mass difference for peptide established as the ratio $|M_{experimental} - M_{calculated}|/Mcalculated \times 10^{-6}$

Table S2. Characteristics of identified kinases in both experiments

Molecular mass	Score	Coverage [%]	Sequences	emPAI	ID	Description	Modyfication
						**Experiment 1**	
44985	6174	72	26	21.47	P00558	Phosphoglycerate kinase 1	N/D
45166	1700	18	6	1.12	P07205	Phosphoglycerate kinase 2	N/D
45229	559	30	8	1.12	P68400	Casein kinase II subunit alpha	N/D
40919	495	23	7	1.06	P55263	Adenosine kinase	N/D
40678	388	20	6	0.87	P17612	cAMP-dependent protein kinase catalytic subunit alpha	N/D
41358	218	9	3	0.36	P19784	Casein kinase II subunit alpha'	N/D
40711	209	13	6	0.68	P22694	cAMP-dependent protein kinase catalytic subunit beta	N/D
42702	186	11	5	0.49	P51570	Galactokinase	N/D
41762	159	13	3	0.36	P28482	Mitogen-activated protein kinase 1	N/D
49972	136	4	3	0.19	Q4G0N4	NAD kinase 2, mitochondrial	N/D
39399	109	5	2	0.24	Q70UQ0	Inhibitor of nuclear factor kappa-B kinase-interacting protein	N/D
53821	65	1	2	0.08	Q9UBS0	Ribosomal protein S6 kinase beta-2	N/D
						**Experiment 2**	
58470	217	8	5	0.24	P14618	Pyruvate kinase PKM	N/D
43183	96	4	1	0.1	P10644	cAMP-dependent protein kinase type I-alpha regulatory subunit	N/D
44985	69	6	2	0.21	P00558	Phosphoglycerate kinase 1	N/D
51242	56	2	1	0.09	P41240	Tyrosine-protein kinase CSK	N/D
41358	45	5	1	0.11	P19784	Casein kinase II subunit alpha'	N/D
						**Indentities of proteins (phosphorylated peptides found only in experiment 2)**	
57169	40	6	1	0.12	A0A0B5HR54	Serine/threonine-protein kinase receptor	Phospho (S)
54871	37	6	1	0.06	Q8NER5	Activin receptor type-1C, Serine/threonine protein kinase which forms a receptor complex on ligand binding. The receptor complex consisting of 2 type II and 2 type I transmembrane serine/threonine kinases. Type II receptors phosphorylate and activate type I receptors which	2 Phospho (S); Oxidation (M)
						**Kinases found in experiment 2 (high uncertainty)**	
153103	3	1	1	0.12	Q99570	Phosphoinositide 3-kinase regulatory subunit 4	Phospho (S)
242676	1	0.7	1	0.11	Q9Y3S1	Serine/threonine-protein kinase WNK2	Phospho (S)

115201	1	2	1	0.05	Q13873	Bone morphogenetic protein receptor type-2, On ligand binding, forms a receptor complex consisting of two type II and two type I transmembrane serine/threonine kinases.	Phospho (2S 1T), Oxidation (M)
469089	1	1	1	0.04	P78527	DNA-dependent protein kinase catalytic subunit	Phospho (S); Oxidation (M)
96984	1	0.3	1	0.06	A0A140VK35	1-phosphatidylinositol 4-kinase	Phospho (ST)
